# Midwives’ psychological experiences following a maternal mortality at a hospital in Eswatini

**DOI:** 10.4102/hsag.v30i0.2976

**Published:** 2025-10-21

**Authors:** Suzan K.M. Mabasa, Zandile S. Khulu, Lily K. Motswasele-Sikwane

**Affiliations:** 1 Department of Nursing, Faculty of Health Sciences, Sefako Makgatho Health Sciences, Pretoria, South Africa; 2 Department of Nursing, Southern Africa Nazarene University, Mbabane, Eswatini

**Keywords:** coping strategies, maternal mortality, maternal death, midwives, psychological

## Abstract

**Background:**

Maternal mortality is a globally recognised health indicator reflecting the quality of maternal healthcare services. Midwives are primary caregivers in maternal health and play a crucial role in reducing maternal mortality. The occurrence of maternal death can have a profound psychological and emotional impact on the well-being of midwives as they provide continuous care and support to expectant mothers and their families. While much of the existing literature focuses on the effects of maternal death on families, limited attention has been given to its impact on midwives.

**Aim:**

This study aimed to explore and describe the psychological impact of maternal deaths on midwives’ well-being.

**Setting:**

The study was conducted in a private room of a maternity ward of a main referral hospital in Eswatini.

**Methods:**

A qualitative exploratory-descriptive research design was used. Ten purposively selected midwives from the maternity ward of a referral hospital participated in individual and 12 in focus group interviews. Data were analysed thematically to identify themes and sub-themes.

**Results:**

Two main themes emerged: Midwives’ negative psychological experiences following maternal deaths, and their recommended strategies for coping with these challenges.

**Conclusion:**

Midwives experienced psychological and emotional distress following the death of women they cared for during pregnancy, labour and the puerperium.

**Contribution:**

The findings of this study could guide employer interventions that strengthen midwives’ coping strategies following a maternal death, thus helping prevent burnout and reduce staff turnover.

## Introduction

Maternal mortality is defined as the death of a woman while pregnant or within 42 days of pregnancy termination, irrespective of the duration and site of the pregnancy, from any cause related to or aggravated by the pregnancy or its management, but not from accidental or incidental causes (National Department of Health [NDoH] 2021). Positive experiences throughout pregnancy, labour and the postpartum period are necessary to ensure that mothers and their unborn children achieve their maximum potential for health and well-being. Sadly, women and their families continue to encounter significant risks during these periods of life, as inadequate health care and accompanying complications claim the lives of women in many areas of the world. The Sustainable Development Goals (SDGs) target calls for a reduction of the global maternal mortality ratio to less than 70 maternal deaths per 100 000 live births by 2030 (World Health Organization [WHO] 2023). All women need access to high-quality care during pregnancy, labour and after childbirth. According to the WHO (2019), the sub-Saharan African region accounted for over two-thirds (65%) of global maternal deaths, with the vast majority (94%) occurring in low- and lower-middle-income countries. Maternal mortality is regarded globally as one of the primary health indicators that show how well healthcare is provided. Globally, maternal health is seen as a significant public health issue, particularly in low- and middle-income nations, which experience the highest rates of morbidity and mortality (Endjala, Amukugo & Nghitanwa 2021).

The Maternal Death Review for 2022 concluded that the Kingdom of Eswatini’s national institutional maternal death rate was 126/100 000 live births (WHO 2023).

Pregnancy-induced hypertension emerged as the leading cause of maternal deaths, contributing to 36% of cases. The review further reported that 36% of maternal deaths were mainly caused by pregnancy-induced hypertension, and other causes included pre-existing diseases such as diabetes, tuberculosis (TB) and human immunodeficiency virus (HIV). Concerning data revealed that almost half of the women attended Antenatal Care (ANC) only once during their pregnancy or were unbooked.

According to Mohale and Nyangu (2021), maternal mortality is a pervasive problem that frequently occurs in developing countries, driven by socio-economic issues, healthcare service-related issues, pre-existing health conditions, health professional-related issues and socio-cultural issues. In a study conducted in Ghana, Stabnick et al. (2022) identified that preventable and treatable complications, including postpartum haemorrhage, infection, hypertensive disorders of pregnancy and unsafe abortion, caused most maternal deaths. All births should be attended by skilled healthcare workers (HCWs) to have better outcomes. However, in most sub-Saharan African countries, there is an unavailability of skilled obstetric providers, including midwives and obstetricians (Stabnick et al. 2022).

In a study conducted by Endjala et al. (2021), after a maternal death was reported, midwives were left with feelings of helplessness, grief and emotional stress. Their psychological well-being was never attended to. Similarly, the study conducted in Ghana revealed that midwives demonstrated emotional distress and self-blame after a maternal death occurrence (Stabnick et al. 2022). Health Care Workers, especially midwives, are the ones who attend to a woman during pregnancy, labour and puerperium and witness complications, such as maternal deaths and near misses. Even though maternal deaths can result from elements outside a midwife’s control, the burden of care can significantly affect their emotions, especially when doubts arise concerning overlooked warning signs or choices made during childbirth. A study by Endjala, Nghitanwa and Hatupopi (2025) revealed that maternal deaths profoundly affected midwives, causing both psychological symptoms, such as depression, sadness, uncontrollable crying and physiological disruptions, such as sleep and appetite disturbances.

A study by Dartey and Ngaiyaye (2020) showed that midwives frequently experienced sleeplessness following a maternal death and that the majority of them were unable to sleep because they were too focused on the incident Midwives’ negative feelings during and after the birth trauma, including shock, sobbing, remorse and helplessness, may have been risk factors for post-traumatic stress disorder (Mofe 2024). These findings align with Endjala et al. (2025), who revealed that midwives displayed denial and sadness following maternal deaths, particularly when adequate support was lacking. Midwives experience significant emotional distress when women under their care die during or following childbirth.

Little attention has been paid to the psychological well-being of midwives after maternal death. In a study conducted by Endjala et al. (2021), following confirmation of a maternal death, midwives were left with feelings of helplessness, grief and emotional stress. Furthermore, midwives experienced post-traumatic stress disorder (PTSD) related feelings, such as insomnia and nightmares, recollection of the event (flashbacks), sense of self-blame, guilt, anger, shame and being tormented after a maternal death (Endjala et al. 2021; Stabnick et al. 2021).

Their psychological well-being was never attended to. It is not clear why the psychological well-being of midwives was never fully explored, as in most studies, the focus is on the family of the deceased only. Midwives might find themselves doubting their skills and reflecting on whether there was anything they could have attempted to avert the death. They may also experience feelings of guilt for not being able to preserve the woman’s life. This prompted the researchers to explore the psychological experiences of maternal deaths on midwives. This study was guided by Betty Neuman’s Systems Model, a comprehensive and dynamic theoretical framework that conceptualises individual midwives as open systems continuously interacting with both internal and external stressors. The model is particularly relevant for examining psychological experiences, as it emphasises the impact of stressors, the individual’s responses to these stressors, and the interventions required to maintain or restore system stability.

## Research methods and design

### Research setting

The study was conducted in a private room of a maternity ward of a main referral hospital in Eswatini, where most of the maternal deaths occurred.

### Study population

According to Gray and Grove (2021), population refers to the group of elements, which could be individuals, objects, events or substances with specific characteristics that are the focus of the study. In this study, the chosen population was midwives from a maternity ward because of their role in managing women during pregnancy, childbirth and postpartum.

#### Inclusion criteria

The inclusion criteria were midwives with more than five years’ experience in a maternity ward with: a bachelor’s degree in nursing sciences and majoring in midwifery, a post-diploma certificate in midwifery, advanced midwifery as a speciality, as well as midwives in management positions.

#### Exclusion criteria

The exclusion criteria were all midwives with less than five years’ experience in the maternity ward and working in other wards.

### Sampling

A purposive sampling was used to select midwives working in the maternity unit, as this meant that they could purposefully inform an understanding of the research problem and central phenomenon in the study.

#### Sample size

The sample size consisted of 10 midwives from a maternity ward who were interviewed and 12 midwives for the focus group interview. Midwives were chosen for their role in managing women during pregnancy, childbirth and postpartum.

### Data collection

In this study, the researcher collected data using interviews (individual and focus groups), observation and field notes. The interviews took place during the day, behind closed doors, and codes were used to protect the participants. A semi-structured interview guide was developed and used throughout the research, both in the individual and focus group interviews (Khulu 2017). Face-to-face individual interviews were conducted with the nurse managers and advanced midwives and they lasted for 45 min per session. The researcher used an audio tape to record all the interviews throughout the research. The researcher listened intently to each participant, took notes and observed their facial expressions and voice tones throughout the interview (Polit & Beck 2021).

**TABLE 1:** Socio-demographic profile of the participants.

Participant	Gender	Age (years)	Qualification	Work experience in maternity (years)
1	Female	48	Advanced midwifery	10
2	Female	45	Advanced midwifery	13
3	Female	52	Post-diploma certificate in midwifery Bachelor’s degree in nursing education Master’s degree in public health	25
4	Female	50	Post-diploma certificate in midwifery Diploma in Child Health Sciences Bachelor’s degree in Medical and Surgical Nursing and Community Mental Health	8
5	Female	30	Bachelor’s degree in Medical and Surgical Nursing and midwifery	6
6	Female	30	Post-diploma certificate in midwifery	7
7	Female	33	Bachelor’s degree in Community Health Nursing and Midwifery	6
8	Female	31	Post-diploma certificate in midwifery Bachelor’s degree in Community Health, Administration and Management	8
9	Female	31	Post-diploma certificate in Midwifery	6
10	Female	31	Post-diploma certificate in Midwifery Bachelor’s degree in Community Health and Education	6
11	Female	36	Bachelor’s degree in Community Health and Midwifery	10
12	Female	47	Bachelor’s degree in Medical and Surgical Nursing and Midwifery	8

For the focus group interviews, the researcher interviewed four to six midwives at the same time. The questions asked of the participants were open-ended and addressed to the whole group. Data saturation was achieved through five interviews.

### Data analysis

The researcher used Tesch’s eight-step process for qualitative data analysis (Creswell 2013). Field notes were made, an audiotape recorder was used and the data were verbatim transcribed. Triangulation was accomplished by systematically comparing and integrating the data collected from field notes, observations and face-to-face interviews. Emerging themes from the interview transcripts were cross-checked with observational data to ensure consistency, identify patterns and enhance the credibility of the findings. Coding was carried out to categorise and group similar information into categories and later into themes. The researcher and the co-coder met after the analysis to reach a consensus.

### Trustworthiness

Trustworthiness of the current research was accomplished through credibility, confirmability, transferability, dependability and authenticity based on Lincoln and Guba’s (1985) position.

**TABLE 2:** Themes with sub-themes drawn from midwives’ feedback during interviews.

Theme 1: Experiences	Theme 2: Strategies or recommendations
**Sub-theme 1.1: Feelings experienced** Bad, devastated, hurt, failure, painful, depressed, emotionally harassed, bonded with patient, traumatic, confused Empathy or sympathise with family or relatives Blaming self, colleagues, patients and others	**Sub-theme 2.1: For management** In-service training: drills or practice scenarios, forums, refresher courses or workshops Permanent (longer placements in the midwifery unit) Team building: sessions and/or exercises, counseling
**Sub-theme 1.2: Challenges** Resources: human (staff shortages) and other (medication, equipment, building layout) Lack of competency and specialisation in midwifery: inexperience, changeover of staff Emergency readiness Communication and lack of teamwork between health professionals Attitudes of staff, patients and family: laziness, a lack of commitment, patients’ previous experiences Delays in treatment: history taking, misdiagnosis and mismanagement of patients Culture or beliefs of patients and community: teenage pregnancies, use of herbal medications Routine work: paperwork and amount without necessary resources	**Sub-theme 2.2: Nationally** Develop guidelines and/or strategies Providing resources: human and other Give health education via media: television, radio, road shows, pamphlets, also include partners Involve all stakeholders Better infrastructure: transport and/or roads and available or closer clinics Giving more incentives: vacation leave, financially

TV, television.

Credibility was ensured using prolonged engagement and persistent observation as well as triangulation and peer debriefing. Transferability was ensured by using multiple sources, such as individual interviews, focus group interviews, field notes and observation. External audits were used to confirm dependability. Confirmability was ensured through an audit trail, a transparent description of the research design and data collection. The findings of the participants were presented in direct quotations to preserve authenticity and prevent the contents of their experiences from being misrepresented.

### Ethical considerations

The research proposal was presented to the School of Health Care Sciences Research Ethics Committee (SREC), and approval was obtained from the Sefako Makgatho Health Sciences University Research Ethics Committee (SMUREC) and Ethical clearance was granted (SMUREC/H/43/2016:PG). Approval was also obtained from the Ministry of Health (Swaziland), and permission was obtained from the Matron in charge at Mbabane Government Hospital. The following ethical principles of concern that were observed in this study included: privacy, beneficence, justice, anonymity and confidentiality throughout the whole research process. The participants who freely participated were asked to provide written informed consent. The interviews took place behind closed doors, and codes were used to protect the participants’ identities. Only the study was conducted using the safely stored data.

## Results

Two main themes emerged from this study, with sub-themes. This study revealed that after maternal death, midwives feel hurt, depressed, emotional and harassed. They further resorted to blaming themselves, colleagues, patients and others. Themes are outlined below followed by sub-themes.

### Theme 1: Experiences

#### Sub-theme 1.1: Feelings experienced

The midwives commente d that they feel sad and hurt during the events of maternal deaths. The participants’ views were as follows:

‘Yahhh it was a bad feeling.’ (Participant 2)

‘There is a lot of things, we do feel bad.’ (Participant 2)

‘I would say I felt very bad.’ (Participant 3)

‘A maternal death to me it’s hurting …’ (Participant 3).

The midwives described the events as painful and traumatic most of the time because they had already bonded with the patient. Some of these cases are inevitable as the midwives reported hence it is a painful experience. The participants said:

‘Haaaaa ok maternal deaths … it’s traumatic first more than the pain of losing someone.’ (Participant 4)

‘It’s a very traumatic event I would say because it’s like you have a close relationship with the patient that you created. Then after you have provided all the care that you have then suddenly the woman dies.’ (Participant 2)

‘That’s a very painful experience and each time you have one you know that yah its bad.’ (Participant 3)

‘Mmmmm it’s usually very painful because normally they die after you have worked and worked.’ (Participant 3)

The midwives argued that they rarely forget maternal mortality and it is never easy to accept a maternal death:

‘We become depressed. We feel bad, really bad.’ (Participant 1)

‘It’s a depressing experience.’ (Participant 4)

The midwives expressed that they empathise and sympathise with the family or relatives and must provide answers as to how the woman died. They mention that they are always there for the woman’s family and with them during the time of their loss. The participants echoed:

‘I don’t know whether it’s empathy with the family because everyone has lost a member. Sometimes the mother has got other babies.’ (Participant 3)

‘We also sympathise with the relatives. Sometimes we cry together with them.’ (Participant 2)

‘The family is expecting answers, you must comfort them, counsel them, and explain what had happened because the woman came into the ward normally in labour, which is a normal process. No one is expected to die, so when you tell them that the woman is no more, you have to sit down with them and explain what you really did to this woman.’ (Participant 4)

They usually ask themselves what they should have done differently to save their life. Midwives expressed that when they think they have given their all and have tried everything they could, yet the woman still dies, they are blamed by supervisors and other colleagues. Some relatives point fingers at the midwives and want answers as to how their relative passed away in the care of midwives and doctors:

‘So when you are involved in that maternal death you know that you have to account for everything that happened at the same time trying to introspect yourself. Trying to look at what you have done right and done wrong. Some people are expecting a report from you, a thorough report, so it’s traumatic, it’s stressful.’ (Participant 4)

Midwives mentioned that they blame the patients after they have died for delaying coming to the hospital or for the use of traditional medication that has irreversible effects. One participant expressed the blame:

‘To add on that one and take you back a bit, sometimes you blame the patient after the patient has died. In my experience it happened that one lady died, she had drunk this traditional concoction [*Masheshisa*] and then she stayed at home until she came to the hospital fully dilated.’ (Participant 1)

#### Sub-theme 1.2: Challenges

**Resources – shortage of staff:** The midwives attributed most of the maternal deaths to a lack of human resources and other necessary resources. The participants said:

‘The staff shortage.’ (Participant 3)

With the shortage of staff, the midwives complained that they are overworked, which leads to the mismanagement of the clients and burnout. The participants shared their experiences as follows:

‘We have a challenge of staff shortage.’ (Participant 3)

‘With the doctors, we have the general practitioners, but we have a shortage of the specialists. With the midwives, although we don’t have the specialised midwives, the advanced midwives, the midwives that we are having, the number is not enough for the numbers that we deliver.’ (Participant 3)

**Resources – a lack of equipment and supplies:** The midwives also reported that on some days, there is enough manpower, but there is a lack of equipment and supplies to attend to patients. The following quote is evident:

‘Sometimes the deaths are not caused by the fact that you were not enough on duty.’ (Participant 2)

The midwives also reported that there are breakages that are not repaired on time, yet they are necessary for care.

The midwives mentioned that even if they have the knowledge, the skill, and a little experience, they find that there are no necessary drugs and supplies for them to work outstandingly.

**Emergency readiness:** The midwives reported that it is a challenge that the midwifery team lacks emergency readiness and exposure to emergency procedures because they run away when there is an emergency. Some participants said:

‘You find that we are not always ready for an emergency.’ (Participant 1)

**A lack of competence, skills and speciality:** There is a lack of competence, skills and speciality both from the midwives and the doctors. With the available staff, the midwives complained that there is a lack of skill and there is a long routine to be carried out, wherein some patients become neglected. One participant explained:

‘They lack exposure to the emergency procedures because like I said other people will run. Training different people every year becomes tiresome for the midwives and exhausting because maternity is different from all the other departments.’ (Participant 1)

The participants raised their concerns as follows:

‘The short-term ones, like the neonatal resuscitation, some of the nurses did that, but because of the frequent changeover, I have lost some of them to other wards, the skilled nurses.’ (Participant 1)

**Attitude:** The attitude of both midwives and patients plays a major role in contributing to the many maternal deaths, as described by the midwives. Participants expressed the following views:

‘Mhhhh … It’s attitude.’ (Participant 3)

‘Nurses tend to make things that are comfortable to them.’ (Participant 3)

‘Sometimes we have maternal deaths not because the client was poorly managed but because the client has delayed to come to the health facility or the client has not attended ANC at all.’ (Participant 3)

‘But sometimes other patients tell themselves that I will always deliver at home. I will go to the hospital once there is a problem.’ (Participant 1)

‘Then today I felt this headache, this blurred vision then I came to the hospital. I think its lack of sensitisation which I don’t know how we can compensate it. So the patient delay in that sense, also financially. Some will be waiting for the husband from Johannesburg to come with the money so that I can go to the hospital.’ (Participant 4)

The midwives complained that some women keep information away from them yet that is critical for their care and to prevent complications timely. This was expressed as follows:

‘Some things are secret, just like the parity, someone will tell you I am a primup yet she had six abortions because this one is the first one to carry to term then she will say I am a primup.’ (Participant 2)

**A lack of teamwork:** A lack of teamwork among health professionals was reported as a constraint. The participants said:

‘[*W*]ill say that the way we report doesn’t make them wake up fast, then they come, you see …’ (Participant 3)

‘I shouted at that nurse. She is still bitter with me.’ (Participant 3)

‘There is no longer team spirit here like it used to be there.’ (Participant 1)

**Delays in treatment:** Delayed treatment was also reported to be contributing to maternal deaths. The following statements support the views:

‘So if you have misdiagnosed me, so obviously you are not going to be able to manage me accordingly.’ (Participant 3)

‘[*T*]his leads to the mismanagement of clients. There is too much work that they do and some of the things they don’t do properly.’ (Participant 3)

It is evident, as expressed by the midwives, that most of the maternal deaths are preventable, especially when there is no delay and everything that is needed is available. The midwives complained that the constant change of staff contributes to delays in diagnosis and treatment. One participant lamented:

‘The reasons can be many. There might be many people. I could be a shortage of staff and this thing that they put me in maternity, yet I don’t like maternity, it could be my laziness too.’ (Participant 1)

Midwives must practice proper history-taking when attending to patients. The midwives complain that if history is not taken well, the patient will not be treated on time. One participant said:

‘We sometimes, if there was no proper history taking then the management won’t be proper management …’ (Participant 5)

The midwives further complained that doctors tend to give orders without examining the patients themselves, which delays the treatment, especially if there is an information gap:

‘Making a phone order without seeing the patient, not assessed.’ (Participant 1)

The midwives complained that the staff usually take their time in acting on a presented situation of a patient. One participant expressed that:

‘They don’t act promptly.’ (Participant 2)

**Culture or beliefs of patients and the community:** The patients and the communities that they live in have beliefs, and their behaviour contributes to the leading causes of maternal deaths. One participant reported:

‘So, this woman was seen at ANC, she was advised to go to the hospital, but when we asked further, she said she took herbs to decrease the BP.’ (Participant 2)

Some religions believe in avoiding hospitals altogether because God is there to heal illnesses. Midwives mentioned that those patients even deliver at home, where complications occur, and they come to the hospital when it is already too late. So said one of the participants:

‘There are many reasons. Some would state their beliefs that they don’t believe in going to the hospital.’ (Participant 3)

The midwives reported that it is a practised culture in some communities that they have their traditional midwives for all their births. The participant’s comment was:

‘Before we go on, before I forget, there are families in the community who know that when a person is pregnant, when they are in labour, a certain mother will deliver them. It is known, it is the culture of that community, that we deliver our women here and then they go the hospital to get the immunisation card only.’ (Participant 3)

According to the midwives, abortion is still on the rise in Swaziland, yet it is illegal. Women abort, and they encounter complications that lead to their deaths because they usually report to the hospital when it is too late. One participant said:

‘In my case, it was last year it was around March or so. The woman came having delivered at home. Seemingly, the woman was trying to abort, and when you looked at the baby, it was around five or six months or so.’ (Participant 4)

**Routine work:** The routine work that needs to be done and which is outside the monitoring of labour and conducting deliveries, adds to maternal deaths. The participants said:

‘I did but someone told me she was busy with the routine. There is always a routine when there is a critical patient. Even after the patient fell, they said I was busy with the routine.’ (Participant 2)

‘During the day there is a long routine that you do in discharging patients who delivered normally. At the same time, you receive patients who have just been done C/S.’ (Participant 2)

Midwives also observed that the amount of paperwork in maternity was so massive in such a way that it became unmanageable. The participants shared their experiences:

‘We must remember that there is also this paperwork. Sometimes we push the work because I must do this if I don’t do it superiors will come and find things that I have left … Yes work that is not documented is not done.’ (Participant 2)

### Theme 2: Strategies or recommendations

The midwives believe that maternal deaths can be reduced in the hospital if the management and the ministry can do the following:

#### Sub-theme 2.1: For management

**In-service training:** Midwives reported that they have departmental training once a week, where they discuss common and other conditions that they think are important in their care. The participants shared their experiences:

‘I have forgotten something because practice makes perfect. You find that I have forgotten, so maybe if we can have the drills sometimes. Fine, we do have our CME every Thursday so that we can remind each other because it is not for midwives only, even for the doctors.’ (Participant 4)

‘I can learn something from someone who has been there for a long time or someone who has come with new information. Research is continuous there are new things every day. The discussion of the maternal deaths is a good thing on the learning side.’ (Participant 4)

‘Refining our skills.’ (Participant 3)

The midwives agreed that maternal deaths could be reduced and eventually eradicated if midwives could be sent for specialist training. Excerpts from some participants are as follows:

‘Sending people for training.’ (Participant 1)

‘Also having a training plan in mind, especially with our supervisors, because I understand in other units there is a way to train nurses who are interested in that field. If there could be a training plan that this year, we are taking out these nurses for training, whether it’s a refresher, but some kind of training that will come back and help the unit.’ (Participant 4)

Midwives also mentioned that having supervisors who are experienced in maternity and have advanced certificates can improve the quality of care drastically and reduce the staff shortage, hence reducing maternal deaths. One participant said:

‘It would be good to have supervisors who have experience and maybe proper qualifications in midwifery in the labour ward. Not everyone who is promoted can be taken to the labour ward. I think that one can also strengthen the quality of the service in the labour ward. Let’s say this supervisor has got advanced midwifery and she has practised in the labour ward, they take that person to come and supervise me in the labour ward, we are starting to work there.’ (Participant 4)

The midwives believe that refresher courses can be of great help to the staff. The refresher courses or workshops can be on the management of current emergencies and illnesses. The midwives also mentioned that it should not be the same people who attend these workshops. Furthermore, after attending, they should disseminate the information to all staff. Views were as follows:

‘Yah maybe for some time, those refresher courses because the in-service workshops …’ (Participant 3)

‘Refresher courses’ (Participant 4)

**Permanent (longer placements in the midwifery unit):** The midwives complained of the yearly changeover that occurs in the hospital. The proposals are as follows:

‘With the changeover, I would suggest maybe that most of the nurses must stay behind, maybe only 25% can be changed.’ (Participant 5)

‘[*F*]or staff rotation that at least people should be placed in maternity for two to three years, without a change.’ (Participant 4)

‘At least three to five years. For the development of the skill. Than having new ones every year, who do not have the skill, have been out of practice for some time.’ (Participant 2)

‘In my view, I would say they can stay for five or more years. So that they can gain the experience.’ (Participant 3)

The midwives also suggested that during the changeover, the management should place midwives on maternity because of their interest. Additionally, they did not see a reason why a midwife who loves maternity should not work in maternity for the rest of her life unless she has a request to go to another department. The following sentiments were shared:

‘I so wish that people who love midwifery could be the ones that are assigned here, not everybody.’ (Participant 1)

‘My suggestion would be if things went well maybe we would say anyone interested in working in the maternity unit would just be hired for the maternity unit.’ (Participant 3)

‘I don’t see the reason why people shouldn’t stay in one department for their whole lives if they are happy there.’ (Participant 3)

‘We mentioned that a person should be put in a department that she likes, a department of her choice, and she stays maybe for a time that she wants, she goes out per request or after four or five years.’ (Participant 1)

**Team building and counselling:** To reduce maternal mortality, the management should plan for staff motivation, which can be in the form of team-building exercises, so that the midwives have some time out of the ward. Midwives’ views were:

‘Team building exercises maybe. If we can get a psychologist to do that for us because we work as a team.’ (Participant 2)

‘Also, the team building for motivation we need them. If it must be done in a picnic way or whatsoever let it be there but the motivation of the staff, we need it besides money we need the motivation on the staff.’ (Participant 4)

Furthermore, the midwives should be provided with a counsellor to whom they will be able to offload the problems that they face in maternity. The participants lamented:

‘I feel that nurses working in maternity need a counsellor, psychologically.’ (Participant 4)

‘We need a bucket where we can offload.’ (Participant 3)

#### Sub-theme 2.2: Nationally

**Develop maternity guidelines:** The Ministry of Health must take maternity guidelines and policies for the management of complications as a priority for the staff to use to reduce maternal deaths. The participants’ views were:

‘[*W*]ith health personnel factors they say what they found and maybe we must update the protocols and continue to make everyone aware.’ (Participant 4)

‘The problem is the protocols. At times you have to go through certain steps. When doing the recommendations, you write them and take them to this office. When you have written the recommendations you have to take them to office number one, even if you see that office number one is not doing anything, the protocols do not allow you to go to office number two.’ (Participant 2)

**Providing resources – Human and other:** The midwives reported that the Ministry of Health should improve the staffing norms or levels of the hospital for both midwives and doctors. This improvement will help to reduce burnout among the midwives, which then leads to mismanagement of clients, which later results in maternal deaths. Participants expressed their inputs as follows:

‘More staff, incentives.’ (Participant 3)

‘Depending on the setup, I would say the maximum ratio to have is 1:5. You can’t be expected to see and manage more than five patients per shift as a nurse when you are serious with work. I can say I suggest that the maximum ratio be 1:5.’ (Participant 4)

‘To the Ministry of Health via the Matrons and Chief Nursing Officers office. We have also presented the issue of the staffing levels to the senior staff in the Ministry of Health. I am not sure about the date, but it’s July. So, we are still waiting for the response. And we tried to give them rationale why we need these people because in the postnatal ward, for example, the new structure is a semi-private facility.’ (Participant 4)

The Ministry of Health should ensure that more midwives are trained at a speciality level. The notion is supported by the following excerpt:

‘Ok. I think on the staff shortage if our government can hire more experienced doctors and experienced midwives. Besides hiring allow the midwives to pursue their studies in advanced midwifery or degrees in midwifery or just to progress themselves on the midwifery part.’ (Participant 3)

The midwives also complained of the shortage of equipment in the hospital, where at times they are forced to run around before they work, which delays the care. The ministry should, therefore, ensure that the maternity department is a priority in getting all the necessary equipment, drugs and supplies. One participant said:

‘Address equipment, if possible, especially the emergency ones. Let’s try and make sure that they are always there; it must not happen that there is nothing to use when working.’ (Participant 4)

The emergency equipment, drugs and supplies should always be available for use any time there is an emergency. The ministry should also monitor and reinforce the department that is responsible for fixing broken medical equipment so that the equipment is repaired promptly.

**Health education via media:** Provide health education through various media channels including TV, radio, road shows, and pamphlets, and also involve partners. The participants said:

‘Health education.’ (Participant 5)

‘By educating her, giving them the information.’ (Participant 1)

‘Another thing can be health education for the public because some of the things lie on the clients themselves.’ (Participant 5)

Communities and families need to understand both what is expected of a pregnant woman in their care and what responsibilities they have towards her. The partner’s availability at times helps in communicating better with the patient and allaying anxiety where needed. The participants’ views were as follows:

‘Also what we can add is that we need to reinforce on public education pertaining a pregnant woman, not only a pregnant woman, also the public needs to know about pregnancy.’ (Participant 1)

‘The first thing that I see is to try to strengthen the system of giving information to clients. If they cannot come for the health education in the morning, let’s rather get written information to give to them.’ (Participant 3)

‘Having roadshows. We have eye clinic, eye days and what what days whereby you go into the community and hold such campaigns.’ (Participant 4)

**Involve all stakeholders:** The midwives suggested that there should be a strengthening of the team and all stakeholders who are involved in the day-to-day business of the maternity unit to reduce maternal deaths. The participants shared their views as follows:

‘“Involving all the stakeholders.” “Pharmacy.” “The management. Normally when there is a problem here like a patient could not pay, could not afford a drug they were structures like you go to the Administrator she puts a stamp you get the drug.’ (Participant 1)

‘Those that are tendering should involve the stakeholders who are dealing with the things.’ (Participant 3)

‘As a maternity, we are having our theatre, I thought involving them just like we have started now, will motivate them. Involving them in the theatre so that they can also claim the on-calls.’ (Participant 2)

**Better infrastructure:** The midwives emphasised that upgrading and developing primary health care facilities is essential for improving maternal care and reducing mortality. The participants said:

‘I also feel that the structures because you will be talking to this couple here, there is a wall here and the people that side are also hearing our stories. We should at least have privacy so that the patients are open, able to tell me further.’ (Participant 4)

‘The partner was able to communicate better with her.’ (Participant 4)

‘And to alleviate the anxiety because the nurse is not always present with the patient.’ (Participant 1)

**Giving more incentives:** Incentives can help motivate the midwives in maternity and show that everyone is thankful for the hard work that they do. The midwives’ suggestions were as follows:

‘Not looking at the hours that someone is supposed to work, just because we know they are always very busy, at least one day maybe a month to go and rest. Extra from the others.’ (Participant 2)

‘Or else just provide tea, sugar, and some snacks for their tea breaks, every day. They work and they don’t get the time to sit down even to eat. If they have to sit down and eat, they are the ones to see what to do from their own pockets.’ (Participant 2)

‘I think the incentive part would help because at times you find yourself running in that unit then at the end of the day you ask yourself, who comes to say thank you?’ (Participant 4)

The midwives believe that it would be much better if they were paid, especially the advanced midwives, as a form of incentive, because they work harder compared to other departments.

## Discussion

The midwives in this study stated that they experienced sadness when a mother died, to the point where they could recall those situations as if they had occurred recently, even though some of them occurred more than five years since. There is evidence that the work and work cultures of midwives can cause psychological suffering. High-quality maternity care cannot be provided if they continue to work when they are not healthy enough to carry out their clinical responsibilities. This aligns with findings by Bentil et al. (2025), who reported that psychological distress following a maternal death negatively impacts midwives’ physical and professional well-being, often manifesting as sleep disturbances, loss of appetite and heightened anxiety.

The findings of this study showed that because midwives stay with women throughout pregnancy and all stages of labour, they develop a bond with them. Therefore, it is emotionally distressing for these midwives to lose them after putting in so much effort, as they feel emotionally tormented when mothers give birth and then pass away. This aligns with the study by Endjala et al. (2025), which confirms that midwives experience post-traumatic stress disorder (PTSD) related feelings and signs such as insomnia and nightmares, recollection of events (flashbacks), self-blame, guilt, shame, anger and being haunted or tormented when there is a maternal death. Similarly, Abraham et al. (2020) found that midwives often internalised blame following a maternal death, with initial responses characterised by denial, profound grief, emotional distress and sleep disturbances, reflecting the depth of psychological impact experienced.

Most of the time, because they had already developed a bond with the patient, midwives characterised maternal deaths as stressful and painful experiences. According to the midwives, some of these circumstances are unavoidable, which makes them unpleasant. They feel as though they have lost a relative, not just a stranger. Similarly, Mofe (2024) confirmed that following a maternal death, midwives had some of the signs of post-traumatic stress disorder (PTSD), including nightmares, flashbacks, forgetfulness and avoidance. Therefore, it is vital to highlight the experiences and support needs of staff in psychological distress, as there is a clear correlation between staff health and the quality of patient care (Khulu 2017).

The results of this study also showed that most women quietly passed away without showing any symptoms, leaving midwives to bear the pain of witnessing a woman dying in their care. This was described by midwives as sudden deaths, which had a more negative impact on their well-being. This was supported by Morobe, Ramavhoya and Bopape (2025), who found that midwives experienced significant frustration, stress and diminished morale because of a lack of institutional support following maternal deaths. Similarly, McDaniel and Morris (2020) identified midwives as ‘second victims’ of maternal mortality, reporting symptoms, such as burnout, sleep disturbances, depressive episodes, somatic complaints and cognitive stress. One participant stated, ‘none of us (midwives) seem fit’ whenever a maternal death occurred at the workplace. In addition, midwives in this study expressed feeling compelled to act to prevent death but felt puzzled, disheartened and depressed because they were unable to identify what to do in the aftermath of the death (Khulu 2017). Consistent with the study by Endjala et al. (2021), midwives often used self-blame and guilt interchangeably. The findings showed that most midwives blamed themselves, especially after avoidable maternal deaths and recent stillbirths (Endjala et al. 2025).

This study revealed that midwives believe that no woman should die during pregnancy; therefore, when a mother passes away, they remember it for years, which makes them feel melancholic and affects their social interactions. The midwives claim that they hardly ever forget maternal deaths because they are so uncommon, making it difficult to accept them in these situations (Khulu 2017). This was confirmed by Mofe (2024), indicating that when there was a maternal death, it affected midwives negatively as they started having social isolation, meaning that their social relationships at work and home were affected. According to Mohale and Nyangu (2024), midwives resorted to substance abuse and drug abuse when there was a maternal death and engaged in self-medicating.

Certain midwives may feel irate or frustrated, particularly if the death could have been avoided with improved tools, collaboration or structural adjustments. The shortcomings of the healthcare system or procedures that they believe are insufficient in averting such tragedies may frustrate midwives who work in conditions with limited resources or high levels of stress. In this study, midwives had to explain how the woman died while also showing empathy and sympathy for the family or relatives. These fatalities happened without warning, and the midwives are left wondering what might have happened to the mother. Midwives found that they often find themselves crying alongside family members and relatives and must comfort them because maternal mortality is an uncommon occurrence (Khulu 2017). When the woman’s family experiences a loss, they are there for them no matter what, and social support is needed. Several studies demonstrated that while performing diverse routines in health facilities, nurses often face over-commitment and low social support, leading to high levels of stress (Dondonkhuu et al. 2021). This finding is consistent with the study by Hanna et al. (2024), which reported that midwives experienced feelings of helplessness, elevated stress and depression following maternal deaths, ultimately contributing to occupational burnout. Job stress had a direct positive effect on anxiety, and it also exerted indirect positive effects on depression and anxiety through mediating factors.

### Strengths and limitations

The study highlighted the psychological and emotional experiences of midwives regarding maternal deaths. Their experiences were traumatic and led to high levels of stress, and this would make it possible for the employer to apply for extra support, provide trained midwife specialists and improve infrastructure. A notable strength of this study lies in its theoretical grounding in Betty Neuman’s Systems Model, which provided a comprehensive framework for exploring the psychological experiences of midwives following maternal deaths. This framework contributed to an understanding of how stress affects system stability and the need for supportive interventions. Other healthcare professionals, such as doctors and junior categories in nursing, affected by maternal deaths were not included in this study. The study was limited to the psychological experiences of midwives regarding maternal deaths in one hospital, and the results are not a complete reflection of the entire country.

## Conclusion

The study explored and described the psychological experiences of midwives following maternal mortality events in a hospital setting in Eswatini. Findings revealed that midwives experienced significant psychological and emotional distress, particularly when required to report maternal deaths during audit processes, even in cases where the entire clinical team was involved. This reporting responsibility contributed to heightened stress levels, compounding the emotional trauma associated with witnessing maternal death. The study underscores the importance of providing psychological support and counselling services to affected midwives. It further recommends the improvement and expansion of opportunities for midwifery specialisation and the improvement of staffing levels in maternity wards to mitigate the burden on existing personnel.

## References

[CIT0001] Abraham, S.A., Berchie, G.O., Druye, A.A., Prempeh, C.A., Okantey, C. & Agyei-Ayensu, K., 2020, ‘A paradox: Midwives’ experiences of attending a birth resulting in maternal death in a Ghanaian context’, *Journal of Midwifery & Reproductive Health* 8(4), 2447–2455. 10.22038/jmrh.2020.47288.1579

[CIT0002] Bentil, J., Ocloo, V., Atanuriba, G.A. & De Graft, C.B., 2025, ‘“ … we suffer the trauma, yet we soldier on” midwives’ narrations of the effects of maternal deaths on their lives’, *International Journal of Africa Nursing Sciences* 22, 100826. 10.1016/j.ijans.2025.100826

[CIT0003] Creswell, J.W., 2013, *Qualitative inquiry and research design: Choosing among five approaches*, 3rd edn., Sage, Thousand Oaks, CA.

[CIT0004] Dartey, A.F. & Phuma-Ngaiyaye, E., 2020, ‘Physical effects of maternal deaths on midwives health: A qualitative approach’, *Journal of Pregnancy* 2020(2606798), 1–6. 10.1155/2020/2606798 PMC7152977 32308995

[CIT0005] Dondonkhuu, O., Tserenkhuu, L., Nyam, N. & Jurmeddorj, M., 2021, ‘Workplace stress and physiological indices correlation study results’, *Central Asian Journal of Medical Sciences* 7(2), 139–147. 10.24079/cajms.2021.06.009

[CIT0006] Endjala, T., Amukugo, H.J. & Nghitanwa, E.M., 2021, ‘Post-traumatic stress disorder among midwives after exposure to maternal death and stillbirth in Khomas Region of Namibia’, *International Journal of Healthcare* 7(2), 7–14. 10.5430/ijh.v7n2p7

[CIT0007] Endjala, T., Nghitanwa, E.M. & Hatupopi, S., 2025, ‘Psychological impact of maternal deaths and fresh stillbirths on midwives in the Khomas Region, Namibia’, *New Voices in Psychology*, 1–14. 10.25159/2958-3918/17136

[CIT0008] Gray, J.R. & Grove, S.K., 2021, *Burns and Grove’s practice of nursing research: Appraisal, synthesis, and generations of evidence*, 9th edn., Elsevier Inc, St. Louis, MO.

[CIT0009] Hanna, R., Jolanta, B., Katarzyna, B., Kornelia, Z. & Mariusz, J., 2024, ‘The impact of patient death on the risk of developing occupational burnout in midwives a preliminary cross-sectional study’, *Scientific Reports* 14(1), 25634. 10.1038/s41598-024-77607-z 39463420 PMC11514205

[CIT0010] Khulu, Z.S., 2017, ‘Experiences of midwives with maternal mortality at a hospital in Eswatini’, A Dissertation submitted in fulfilment of the degree Magister Curationis in Nursing Science (Advanced Midwifery and Neonatal Science), Sefako Makgatho Health Sciences University.

[CIT0011] Lincoln, Y.S. & Guba, E.G., 1985, *Naturalistic inquiry*, 1st edn., Sage Publications Inc., Newbury Park.

[CIT0012] McDaniel, L.R. & Morris, C., 2020, ‘The second victim phenomenon: How are midwives affected?’, *Journal of Midwifery & Women’s Health* 65(4), 503–511. 10.1111/jmwh.13092 32293795

[CIT0013] Mofe, N., 2024, ‘Exploring the physical and psychological effects of maternal deaths on midwives in a selected hospital of Vhembe District, Limpopo Province’, Dissertation submitted in fulfilment of the requirement for the degree of Master of Nursing, University of Limpopo.

[CIT0014] Mohale, L. & Nyangu, I., 2023, ‘Psychological experiences of midwives regarding maternal deaths at two selected public hospitals in Lesotho’, *New Voices in Psychology* 13, 1–16. 10.25159/2958-3918/15144

[CIT0015] Morobe, R.V., Ramavhoya, T.I. & Bopape, M.A., 2024, ‘Midwives’ experiences regarding adverse events in obstetric units of the selected district of Gauteng province, South Africa’, *African Journal of Reproductive Health* 28(10), 17–27. 10.29063/ajrh2024/v28i10.2 39624942

[CIT0016] Polit, D.F. & Beck, C.T., 2021, *Nursing research: Generating and assessing evidence for nursing practice*, 11th edn., Wolters Kluwer, Lippincott Williams & Wilkins, London.

[CIT0017] South African National Department of Health (NDoH), 2021, *South African national department of health maternal, perinatal, and neonatal health policy*, Government Printers, Pretoria.

[CIT0018] Stabnick, A., Yeboah, M., Arthur-Komeh, J., Ankobea, F., Moyer, C.A. & Lawrence, E.R., 2022, ‘“Once you get one maternal death, it’s like the whole world is dropping on you”: Experiences of managing maternal mortality amongst obstetric care providers in Ghana’, *BMC Pregnancy and Childbirth* 22, 206. 10.1186/s12884-022-04535-z 35287601 PMC8919901

[CIT0019] World Health Organization (WHO), 2019, *Maternal mortality: An evidence brief*, World Health Organization, Geneva, viewed 01 September 2025, from https://coilink.org/20.500.12592/79fk2w”COI:20.500.12592/79fk2w.

[CIT0020] World Health Organization (WHO), 2023, *2022–2023 Eswatini biennial report*, WHO Eswatini Country Office, Mbabane.

[CIT0021] World Health Statistics, 2023, *Monitoring health for the SDGs, Sustainable Development Goals*, World Health Organization, Geneva.

